# Lessons learned from the promotion of Essential Emergency and Critical Care in Tanzania – a qualitative study

**DOI:** 10.1136/bmjopen-2024-089229

**Published:** 2025-10-29

**Authors:** Aneth Charles Kaliza, Linda B Mlunde, Carl Otto Schell, Karima Khalid, Hendry Sawe, Elibariki Mkumbo, Andrew Kigombola, Isihaka Mwandalima, Erasto Sylvanus, Said Kilindimo, Edwin R Lugazia, Janeth Stanslaus Masuma, Tim Baker

**Affiliations:** 1 Department of Emergency Medicine, Muhimbili University of Health and Allied Sciences, Dar es Salaam, Tanzania; 2 Department of Community Health, Muhimbili University of Health and Allied Sciences, Dar es Salaam, Tanzania; 3 Department of Global Public Health, Karolinska Institutet, Stockholm, Sweden; 4 Center of Clinical Research Sörmland, Uppsala University, Eskilstuna, Sweden; 5 Department of Medicine, Nyköping Hospital, Nyköping, Sweden; 6 Department of Anesthesiology and Critical Care, Muhimbili University of Health and Allied Sciences, Dar es Salaam, Tanzania; 7 Department of Anesthesiology and Critical Care, Muhimbili Orthopedic Institute, Dar es Salaam, Tanzania; 8 CIHEB Tanzania, Dar es Salaam, Tanzania; 9 UNICEF Tanzania, Dar es Salaam, Tanzania; 10 Department of Emergency and Clinical Care, United Republic of Tanzania Ministry of Health, Dar es Salaam, Dar es Salaam, Tanzania; 11 World Health Organization Tanzania, Dar es Salaam, Tanzania; 12 Department of Clinical Research, London School of Hygiene and Tropical Medicine, London, UK

**Keywords:** Patients, Emergency Service, Hospital, Health Services

## Abstract

**Objective:**

To describe the lessons learnt during the promotion of a new approach to the care of critically ill patients in TanzaniaEssential Emergency and Critical Care (EECC).

**Design:**

A descriptive qualitative study using thematic analysis of structured interviews.

**Setting and participants:**

The study was conducted in Tanzania, involving 11 policymakers, researchers and senior clinicians who participated in the promotion of EECC in the country.

**Findings:**

Five inter-related themes emerged from the promotion of EECC in Tanzania: (1) early and close collaboration with the government and stakeholders; (2) conduct research and use evidence; (3) prioritise advocacy and address misconceptions about EECC; (4) leverage events and embed activities in other health system interventions; and (5) employ a multifaceted implementation strategy. The themes map to the normalisation process theory domains of coherence, cognitive participation, collective action and reflexive monitoring.

**Conclusion:**

The integration of EECC into Tanzania’s health policy is a result of a multidisciplinary collaboration including government and partners that has used evidence, advocacy and context and included multifaceted implementation strategies. The lessons from Tanzania’s experience provide guidance for adoption in similar settings to improve critical care systems, foster access to care and optimal outcomes for all critically ill patients.

**STRENGTHS AND LIMITATIONS OF THIS STUDY**

- High credibility of findings due to the in-depth qualitative data collection process and the inclusion of diverse participants, with in-depth explorations until data saturation was reached.
- Iterative sharing of findings with participants to ensure the findings align with lived experiences.
- Absence of transcription and translation of the interviews; instead, a codebook and audio recordings were used for cross-referencing, which may have led to some relevant information being missed.
- Some individuals who might have provided additional perspectives were not included in the study, limiting the diversity of viewpoints and experiences that could be explored.
- Participants may have altered their reflections to align with what they perceived to be desirable to the interviewer.

## Introduction

 Illness can progress in severity^1^ if appropriate care is not provided. Progression to the most severe stage of any acute illness, regardless of a patient’s age, gender, social status or medical condition, is termed ‘critical illness’.^2^ Critical illness is characterised by vital organ dysfunction, a high risk of imminent death if care is not provided and the potential for reversibility.^3^ Critical illness is strikingly common—approximately 1 in 10 patients either arriving to hospital or being treated in hospital are critically ill.^4^,^6^ Globally, it is estimated that 45 million adults become critically ill each year.^7^ While the burden of paediatric critical illness is unknown,^8^ sepsis and pneumonia are the leading causes of children’s mortality and 7 million children require oxygen for pneumonia each year.^9 10^ The need for improving the care of critical illness globally and especially in low-resource settings is increasingly recognised.^11^,^13^

In Tanzania and other low-income countries, the burden of critical illness is high, yet health systems often lack the capacity to provide timely and effective care for critically ill patients. More than 90% of critically ill patients are cared for in general hospital wards, rather than specialised units.^14^ Healthcare providers in these wards frequently are not capacitated to identify deterioration of patients early and provide essential life-saving interventions.^15^ Studies from similar settings have shown that these critically ill patients do not receive basic, life-saving supportive care early enough and there is a large burden of preventable deaths subsequently.^16^

To systematically improve care for critically ill patients, a focus has recently been proposed on Essential Emergency and Critical Care (EECC)—the care that should be provided to all critically ill patients in all hospitals in the world.^1^ The EECC approach is guided by three principles: (1) priority to those with the most urgent clinical need; (2) provision of life-saving treatments that support and stabilise failing vital organ functions when indicated; and (3) care to focus on effective actions of low cost and low complexity.^1^ The COVID-19 pandemic, despite its devastating consequences, was a valuable source of learning for health systems as weaknesses were unveiled in the existing critical care model and the need for a more sustainable, inclusive and equitable critical care system.^17 18^ There are substantial gaps between the needs of critically ill patients and the availability of fundamental critical care in health systems in low- and middle-income countries.^16 19^ EECC is an approach that could close these gaps, by focusing the human and other resources in healthcare towards the patients with the greatest clinical need—in line with a key goal for health systems of prioritising resources to maximise health benefit.^20^ The low costs of the 40 simple clinical processes contained in EECC, when compared with advanced critical care such as intensive care, enable care provision to many, even when resources are limited and suggest that prioritising EECC would be an equitable and cost-effective approach.^21^ EECC is also important in settings of higher resources—to ensure that specialised medical services do not overlook foundational care.^2^ The concept of EECC has the potential to improve critical care in hospitals all over the world and substantially reduce preventable mortality.^2^

In recent years, the Tanzanian government has been building a policy emphasis on the care of critically ill patients, with increased momentum due to the COVID-19 pandemic. Researchers have responded with the generation of evidence around critical illness and critical care in Tanzania^22^,^24^ and the Tanzanian Ministry of Health has developed a National Strategic Plan for EECC Services.^25^ The experiences of the introduction and promotion of EECC in Tanzania have provided insights into the challenges, opportunities and potential solutions when introducing and promoting a new perspective to care, EECC. This study aimed to explore the barriers and facilitators in this process as lessons learnt that can guide researchers, policymakers, healthcare providers and organisations involved in improving the care of critically ill patients worldwide.

## Methods

### Study design and setting

We employed a qualitative descriptive design, conducting in-depth interviews with key informants in Tanzania from February to May 2023. Tanzania is a lower-middle income country in East Africa with a population of 65 million people,^26^ a life expectancy of 67 years and an infant mortality rate of 33 deaths per 1000 live births. The country is a multi-party democratic republic with a long history of political stability.

### Study participants

Participants were purposefully selected based on their known contribution to the work around EECC in Tanzania. They included national-level researchers, healthcare professionals, policymakers and representatives from implementing and development partners. All individuals who were approached participated in the study. During interviews, some participants suggested additional key informants involved in the promotion of EECC. The sample size was determined by the principle of saturation, when additional interviews were no longer expected to lead to new data.

### Data collection

Interviews were conducted in both English and Swahili, guided by a semi-structured interview guide (online supplemental appendix). The guide encompassed questions pertaining to the strategies employed, stakeholders involved, facilitators and challenges encountered by participants during the promotion of EECC. The interview guide was piloted and adjusted before use.

The interviews took place either at the participants’ workplaces or via online calls for those inaccessible due to logistical constraints and involved the interviewers and participants only. Each interview lasted between 45 and 60 min and prior to each interview, verbal consent was obtained from the participants. All interviews were audio recorded for accuracy and analysis purposes with field notes taken during each session. All interviews were carried out once; no repeat interviews were conducted. The interview sessions were led by two researchers (ACK and LBM), both possessing backgrounds in clinical medicine and qualitative research methodologies, and with pre-existing professional collaborations with some of the participants.

### Data analysis

Following data collection, ACK and LBM immersed themselves in the interview data to enhance familiarity with the content by repeatedly listening to the audio recordings. Transcription was not possible due to logistical constraints. Analysis was conducted using a rapid qualitative analysis approach.^27^ A deductive approach was employed to develop a codebook based on the interview guide questions. Researchers listened to the audio recordings and manually organised the content of the interview data into predefined codes derived from the codebook. The codes were grouped into categories, from which overarching themes were identified. The emerging themes were discussed among the researchers and then shared with participants for their feedback to ensure their accuracy and resonance with their experiences. The identified themes were mapped to the domains in the normalisation process theory (NPT) framework which focuses on understanding how interventions become routinely embedded in practice.^28^

### Ethical considerations

Participants were provided with information about the study’s purpose, procedures and the interview guide prior to the interview. Verbal consent was obtained from each participant, ensuring they clearly understood the study, freely chose to participate and had an opportunity to ask questions and clarify any concerns before providing their consent. All collected data was securely stored and was only accessible to ACK and LBM. All personal identifiers were removed, and data were anonymised before analysis.

### Patient and public involvement

Patients or the public were not involved in the design, or conduct, or reporting, or dissemination plans of our research.

## Findings

11 key informants were included, including researchers, implementation and development partners, policymakers and emergency and critical care healthcare professionals (table 1).

**Table 1:** Participant characteristics

Characteristics	Frequency n (%) N=11
Female	3 (27)
Age (median, (range))	45 (30–60)
Postgraduate education	9 (80)
Years of professional experience (range)	3–30
Profession	
Researchers	4 (36)
Emergency physicians	2 (18)
Critical care physicians	4 (36)
Policymakers	6 (54)
Implementation partners	4 (36)

Note the percentages add up to more than 100% as several participants had more than one profession.

Five themes emerged from the data as lessons learnt from the promotion of EECC in Tanzania: (1) early and close collaboration with the government and stakeholders; (2) conduct research and use evidence; (3) prioritise advocacy and address misconceptions about EECC; (4) leverage events and embed activities in other health system interventions; and (5) use a multifaceted implementation strategy.

### Theme 1: Early and close collaboration with the government and stakeholders

Participants stressed the importance of early and continuous policy contact, fostering government leadership, plus contact with in-country stakeholders, United Nations agencies and other partners. The EECC agenda was repeatedly communicated to policymakers and other stakeholders to maintain focus and conducting periodic dissemination meetings kept them informed of their research agenda and ongoing activities for the promotion of EECC.

… we did dissemination to get more people to understand the idea better. We invited people from [Universities], [government ministries], health facilities included in the study and other organizations. We introduced EECC to them, and they were very keen to make sure EECC is working. (Researcher, Female, mid 40s)

For any country, the government has many agendas and areas that they are working on improving. To maintain focus on EECC, participants expressed that having an EECC expert based in-country was necessary for continuous contact with the government and stakeholders.

…it is important to establish early and continuous policy contact, for instance for the case of Tanzania, continuous contact about EECC with the Ministry of Health. (Researcher, Male, early 50s)

Additionally, establishing and maintaining close contacts with partner organisations such as UNICEF helped to mobilise resources needed for promotion of EECC and the development of a national strategic plan for EECC.

… [funding] to the government in Zanzibar to do the first phase to devise the plan on how Zanzibar would increase EECC and also a lot of support and engagement with the government led to the National Strategic Plan for EECC… (Researcher, Male, early 50s)

### Theme 2: Conduct research and utilize evidence

Participants emphasised the importance of conducting research to generate knowledge as a key strategy for promoting EECC. Researchers in Tanzania conducted baseline surveys to understand the gaps in the provision of critical care in Tanzania and neighbouring countries; specifically mentioned was research in the provision of oxygen and availability of resources in facilities for the provision of EECC. The findings were disseminated to inform stakeholders about the need for EECC. Furthermore, health economics research to describe the potential cost and cost-effectiveness of EECC was found to be of particular interest to policymakers.

…we conducted baseline surveys to understand the status of critical care such as the critical care in low-income countries study done in 2009 which showed a lack the basic care and equipment for patients in hospitals… (Researcher, Female, mid 40s)

…what we found is mostly what is needed might be present in the facility, but is not at the right place, and maybe not in the right amount.… (Researcher, Female, mid 40s)

Participants acknowledged the need to use the output of research work to inform the government and stakeholders to influence change in the provision of critical care.

…research is not confined to knowledge generation, so it is important to develop an interface between research and policy, research and impact……ultimate job of a researcher is not just to create new knowledge, but to make the world a better place… (Researcher, Male, early 50s)

…We have background information regarding the usefulness of EECC, which I think is one step good, and some conversation with the government regarding implementation of this which has already started… (Researcher, Male, early 50s)

### Theme 3: Prioritize advocacy and address misconceptions about EECC

Advocacy emerged as a prominent theme during interviews with the informants. Participants highlighted that while working to influence decisions, policies and practices in favour of EECC, there were four areas to address to avoid misconceptions:

The significance of clearly communicating what EECC is and ensuring its principles are well understood. For instance, despite being a novel approach to critical care, the set of actions and treatments included in EECC are known practices in caring for sick patients. Additionally, it was found important to communicate that there are gaps in provision of EECC in both low and high-resource settings and that EECC is feasible in both settings and can be administered by any healthcare provider, regardless of their location within the hospital.

…It’s a systems innovation, explaining the problem in a different way than it has been done before, providing a solution in a different way than it has been done before. It should be advocated everywhere in the world… (Critical care specialist, Male, early 50s)

Given that EECC embodies the most foundational processes in provision of care, there is a risk of it being overlooked and undervalued. Participants emphasised the need for clarity in this area while advocating for EECC.

…There was this notion that this [EECC)] is already done, that it is not something that you should be talking about, people were dismissing it. They were like, we know that it is part of what we teach every day…But from experience, from what we have been looking into, from the study, we know that this is not happening all the time… (Policymaker, Male, early 40s)

The provision of EECC entails the reallocation of resources already present in the majority of hospitals, rather than necessitating additional resources.

…A study was done that showed that this basic equipment is present in the hospitals but is either locked away or the person in charge is not around so they cannot be accessed… (Researcher, Female, mid 40s)

…we can make a difference in the outcome of our patients, we do not need to be rich to do that we have all the resources to make the difference, and EECC is trying to prove that… (Emergency physician, Male, early 50s)

The terms ‘critical care’ and ‘intensive care’ are frequently used interchangeably in the health system, and it was not widely understood that critical care can be provided at a low cost anywhere in the hospital, rather than just in intensive care units (ICUs).

…for critical care, people understood that only particular staff should provide it, those in ICU and the emergency department. But even when you work in the outpatient department you should be able to identify a critically ill patient and manage him/her. They thought that when you have a critically ill patient you must send him/her to ICU… (Implementation partner, Female, early 30s)

…every time we are trying to get our message across it’s actually two messages. We must focus on critical illness, and we have to focus on the basics… if the world only hears the first one, that’s a challenge… (Researcher, Male, early 50s)

…people should understand that even if you work in the ward or outpatient department, sometimes you can come across a critically ill patient, and you should be able to initiate something before even those who you regard can take care of that patient take over the management of that patient… (Policymaker, Male, early 40s)

### Theme 4: Leverage events and embed activities in other health system interventions

Participants explained how the increased global attention on emergency and critical care after the COVID-19 pandemic was harnessed to promote EECC. Additionally, they leveraged the focus in international health direction by the WHO for health emergency preparedness, response and resilience during and after the pandemic.

…COVID-19 forced us to look at critical care with our own two eyes. After recognizing that patients need oxygen and we do not have oxygen, and for one to provide oxygen one needs nasal prongs, masks, and gas tanks which were not there, we even realized that we do not have good production of oxygen… (Critical Care specialist, Male, late 50s)

Participants pointed out that they actively engaged with various technical working groups initiated by the Tanzania Ministry of Health in the COVID-19 response and had the opportunity to describe how EECC could enhance the emergency preparedness of health systems.

…we contacted people in the Emergency Preparedness committee to give our thoughts on the drafted guidelines on the treatment of COVID-19… (Researcher, Male, early 50s)

…were invited to the Technical Working Groups during COVID-19 time, when they were preparing COVID-19 treatment guidelines… we started advocating for EECC telling them there would be many critically ill patients who would not benefit from advanced care… (Researcher, Female, mid 40s)

Participants explained that it was crucial that EECC was framed as a stepping-stone towards delivering optimal care and that it complemented already established processes in the health system and initiatives for improving care such as emergency obstetrical care, Emergency Triage and Treatment of children, scaling up of emergency and ICUs and scale-up of oxygen ecosystems; facilitating such initiatives to be more effective and impactful.

…for example, when someone holds a training on malaria and they are explaining when we have a sick child with malaria, we must give antimalarial drugs, but we must also give EECC… (Researcher, Male, early 50s)

### Theme 5: Use a multifaceted implementation strategy

Participants said that employing a multifaceted approach is important when introducing a new concept like EECC. They felt that single interventions (such as a training course, or the procurement of some equipment) are unlikely to have an impact or lead to sustainable change. They further noted that change required engagement from all levels of the health system; from the national Ministry of Health to regional and district-level leaders, to hospital administrators and frontline providers. They viewed this broad-based engagement as essential for driving policy change and supporting the development of Tanzania’s National Strategic Plan for EECC.

… to change policies and strategies, you need a countrywide, system effort to change and implement, from the top, Ministry, to regional local administration, to hospital administrators and providers, all at once… (Emergency physician, Male, early 50s)

Participants’ reflections suggested that EECC implementation was not a linear process but rather an adaptive one, requiring flexibility, persistence and a range of strategies tailored to different stakeholders and contexts.

It is hard to make a system change, it needs a lot of processes, it needs patience, a lot of steps, advocacy, stakeholders buy-in, you have to try to find different ways and approaches to reach to your goals, it does not happen in a short time. (Critical Care specialist, Male, late 50s)

### Mapping the themes to the normalisation process theory

The five themes mapped onto the four domains of NPT: coherence, cognitive participation, collective action and reflexive monitoring as described in (table 2).

**Table 2:** Mapping the themes to the normalisation process theory

NPT domain	What it means for EECC	Relevant theme	Relation between domain and theme
Coherence	How stakeholders made sense of EECC and developed a shared understanding of its importance in improving critical care in Tanzania.	Theme 2: conduct research and use evidence.	Evidence from research showed the gaps in basic critical care and EECC as a feasible and cost-effective solution.
		Theme 3: prioritise advocacy and address misconceptions about EECC.	Advocacy work clarified issues around EECC that would otherwise hinder its uptake with stakeholders or cause it to be mistaken for intensive care.
Cognitive participation	How commitment, involvement and partnerships were built with stakeholders to drive EECC implementation	Theme 1: early and close collaboration with the government and stakeholders	Periodic dissemination meetings, frequent contact with stakeholders and structures for continuous collaboration maintained the focus and ongoing engagement with stakeholders.
Collective action	How EECC was practically implemented into existing health system structures.	Theme 4: leverage events and embed activities in other health system interventions.	Integration with COVID-19 response committees, engagement with technical working groups and framing EECC as complementary to ongoing initiatives.
Reflexive monitoring	How participants evaluated the effectiveness of EECC implementation strategies and identified areas of improvement.	Theme 5: use a multifaceted implementation strategy.	Using many different implementation strategies together provided participants with a broad perspective to evaluate the implementation process.

EECC, Essential Emergency and Critical Care; NPT, normalisation process theory.

## Discussion

We have found that a comprehensive approach including a multidisciplinary collaboration among a diverse group of professionals, policymakers and implementation partners alongside employing multiple strategies, was able to successfully introduce and promote EECC in Tanzania. This approach ensured that interventions synergistically reinforced the others’ impacts and eventually led to the assimilation of the concept, the development of a National Strategic Plan for EECC Services and the increase in activities around EECC in the country. Such strategies that combine efforts to address various barriers have been seen to be more effective than interventions targeting one obstacle at a time,^28^ but can demand additional resources and effort.^28^ It is important to note that participants described the advocacy and implementation of EECC as a complex and system-wide process. Its cross-cutting nature, the diversity of stakeholders involved and the need to shift long-standing norms in critical care made EECC advocacy challenging. This required ongoing coordination across different levels of the health system and active efforts to engage, align and sustain the commitment of a wide range of stakeholders. Acknowledging complexity and tailoring approaches to address specific barriers, as done in Tanzania, has been recommended as a core principle of implementation science.^28 29^

Capitalising on the global emphasis on critical care in the COVID-19 pandemic offered a unique opportunity to promote EECC. Despite the efforts to improve ICUs during the pandemic, most of these units faced major shortages of human resources and medical devices, jeopardising both patients and healthcare workers’ safety.^30^ Viewed as a stress test, the pandemic served as an opportunity to design more effective interventions to maximise scarce healthcare resources.^31^ Based on this, an important research priority has been to develop feasible ways to identify early those who are critically ill, and those at risk of mortality.^31^ The core aspects of EECC are the identification and care of the critically ill, and the provision of life-saving supportive care that is of low cost and low complexity.^1^

When promoting EECC, misconceptions need to be addressed. The novelty of the EECC concept, with its cross-cutting prioritisation of illness severity across all diagnoses, its emphasis on the most foundational care and its system lens, can be at odds with traditional approaches in health systems, leading to significant misunderstandings. EECC is care for critical illness, but it is not ICU care. The clinical processes for the provision of EECC are already familiar to healthcare professionals, potentially leading to confusion about its distinctiveness compared with existing approaches. The absence of a universally agreed-upon definition of critical care further amplifies misconceptions about EECC and its relationship to intensive care. The Tanzanian team, in their advocacy endeavours, placed a strong emphasis on explaining what EECC entails and what it does not, recognising this as the foundation for dispelling misconceptions. The EECC approach prioritises life-saving supportive care across all medical specialties, aiming to complement specialty-based care and bridge the quality gap between current practices and best-practice guidelines.^1^ It is important to clarify that EECC is not advanced care, does not encompass the definitive care of underlying diagnoses, nor intend to encompass all aspects of patient care.

The NPT framework provides a useful tool for understanding the themes. The first NPT domain, coherence was demonstrated through the research and advocacy activities that were conducted such as the process of clarifying EECC’s distinctiveness from intensive care and establishing a shared understanding of its value as a low-cost, life-saving approach to the care of critically ill patients. Cognitive participation was evident in how stakeholders, from policymakers to frontline clinicians, were actively engaged to take ownership and drive the integration of EECC into the health system. Collective action was reflected in the coordinated efforts that brought together multiple strategies and stakeholders to operationalise EECC when required due to the context of the COVID-19 pandemic. And reflexive monitoring was demonstrated as multiple implementation strategies were used based on the needs of stakeholders. Successful implementation of EECC in other contexts is likely to need efforts that include all of the NPT domains.

### Strengths and limitations

The strength of this study lies in the methodology of data collection which involved qualitative in-depth exploration until saturation was achieved during interviews. This method facilitated a profound understanding of the barriers and facilitators for EECC implementation, thereby enhancing the credibility of the findings. Second, the evolving findings were iteratively shared with study participants for their feedback to ensure that what was reported aligned with their lived experiences. Additionally, the professional diversity of the study participants yielded a wealth of material indicative of diverse perspectives. The main limitation of the study was the absence of transcription and translation which could have led to a loss of information even though the code book and audio recordings were frequently revisited. Another limitation was that some individuals who might have provided additional perspectives were not included in the study, limiting the diversity of viewpoints and experiences that could be explored. Lastly, participants may have altered their reflections to align with what they perceived to be desirable to the interviewer.

## Conclusion

The integration of EECC into Tanzania’s health policy is a result of a multidisciplinary collaboration including government and partners that used evidence, advocacy and context and included multifaceted implementation strategies. The lessons from Tanzania’s experience provide guidance for adoption in similar settings to improve critical care systems, foster access to care and optimal outcomes for all critically ill patients.

## Supplementary material

10.1136/bmjopen-2024-089229 online supplemental file 1

## Data Availability

Data sharing not applicable as no datasets generated and/or analysed for this study.
