# Supplementary material for: Lessons learned from the promotion of Essential Emergency and Critical Care in Tanzania – a qualitative study
Source: BMJ Open. 2025 Oct 29;15(10):e089229. doi: 10.1136/bmjopen-2024-089229 (PMC12574400; doi:10.1136/bmjopen-2024-089229)
Supplement: online supplemental file 1 [file bmjopen-15-10-s001.docx]

# **APPENDIX: INTERVIEW GUIDE**

**Muhimbili University of Health and Allied Sciences.**

Greetings!

We would like to welcome you to a discussion about Essential Emergency and Critical Care (EECC) in Tanzania. You have been identified as one of the key stakeholders who has worked on promotion of Essential Emergency and Critical Care in the country. We would like to hear your experience of the process, what has been achieved and how.

Questions

1. When did you first hear about EECC? Was it a new concept to you?
2. In your opinion, why should we advocate for EECC in Tanzania?
3. Were there pushing factors to introducing EECC in Tanzania? For example, External/Societal (e.g. media, advocacy groups/quality)

Probe: what led you to start the efforts of introducing EECC at the time you started?

1. Which strategies have been used to introduce EECC in Tanzania?

Probes: Which activities did you do? How was the process?

1. Whom did you first approach to try to introduce the concept of EECC in Tanzania? What was their response? Who were the other people you approached? How did they respond?

- Did these people know about EECC prior?
- Was EECC found to be acceptable, appropriate, feasible by these people? Please explain.
- In your opinion, why do you think they were able to prioritize EECC than other health care needs?
- Were they ready to adopt, implement, and sustain it? How?

1. Did you have to make partnerships and connections to introduce EECC in Tanzania? What/who were they?
2. What were the economic, environmental, political, technological conditions that enabled you to introduce EECC in Tanzania?

Probes: Was the process funded? Were there any policies and laws that supported the process? Were there any other facilitators for the process?

1. Which challenges did you encounter in the process of introducing EECC in Tanzania?

Probe: What did not go well?

1. What were the successes of your efforts?

Probes: What went well?

1. Were there any unintended outcomes during the process of introducing EECC in Tanzania? What were they?
2. What have you learned in the process of promoting EECC in Tanzania?
3. In your opinion, what is the status of EECC?

Probe: What is going on with regards to EECC now?

1. In your opinion, what are the next steps regarding improving coverage of EECC in Tanzania and beyond?

Probe: What do you envision to see as the future of EECC in Tanzania?

1. If you were to participate in the introduction of EECC in Tanzania all over again, what would you have done differently?
2. What recommendations do you have for others who want to introduce the concept of EECC in their settings?
3. Who else did you work with to promote EECC in Tanzania?
4. Is there anything else that you would like to share with us regarding EECC in Tanzania?

Thank you so much for your time!
