# Supplementary material for: Lessons learned from the promotion of Essential Emergency and Critical Care in Tanzania – a qualitative study
Source: BMJ Open. 2025 Oct 29;15(10):e089229. doi: 10.1136/bmjopen-2024-089229 (PMC12574400; doi:10.1136/bmjopen-2024-089229)
Supplement: Supplementary file 2 [file bmjopen-2024-089229.reviewer_comments.pdf]

PEER REVIEW HISTORY

BMJ Open publishes all reviews undertaken for accepted manuscripts. Reviewers are asked to complete a checklist review form and are provided with free text boxes to elaborate on their assessment. These free text comments are reproduced below.

ARTICLE DETAILS

Title (Provisional)

IMPROVING THE CARE OF CRITICALLY ILL PATIENTS: LESSONS LEARNED FROM THE PROMOTION OF ESSENTIAL EMERGENCY AND CRITICAL CARE IN TANZANIA: A QUALITATIVE STUDY

Authors

Kaliza, Aneth Charles; Mlunde, Linda B; Schell, Carl Otto; Khalid, Karima; Sawe, Hendry; Mkumbo, Elibariki; Kigombola, Andrew; Mwandalima, Isihaka; Sylvanus, Erasto; Kilindimo, Said; Lugazia, Edwin R.; Masuma, Janeth Stanslaus; Baker, Tim

---

VERSION 1 - REVIEW

**Reviewer**	**1**
**Name**	**Nydahl, Peter**
**Affiliation**	**University Hospital of Schleswig-Holstein, Nursing Research**
**Date**	**12-May-2025**
**COI**	**None**

Dear authors,

thank you very much for the opportunity to review the submitted manuscript. The manuscript includes a qualitative study evaluating an improvement project in Tanzania.

I am appreciating this well-written work and have got some minor concerns.

Note to the editors: this manuscript has been submitted to the journal on May 27th 2024 – nearly one year ago, and I reviewing it for the first time on May 12th 2025. What is going on in the BMJ??? Why does it take more than one year for a review?

MINOR CONCERNS

Page 5 (Abstract), Line 24: since this is a qualitative research, please use the term “Findings” instead of “Results”; and in Conclusions “the findings from this study …”; and in main manuscript.

Page 7, Data analysis: please add a reference and method to this paragraph.

P12: in the first paragraph in section Discussion, the design, method, and main findings are summarized. The discussion incl references etc. starts in the second paragraph.

Thank you!

Reviewer 2

Name Lin, Yanxia

Affiliation Shanghai University of Tradition Chinese Medicine, School of Nursing

Date 23-Jun-2025

COI None

Thank you for the review invitation. My comments on the paper are listed below.

1. Title: I think it is too long, with two colons. Remove "Improving the care of critically ill patients:"
2. Abstract: I cannot agree with the authors' Conclusions on the Results that it is a "holistic, comprehensive approach" because the relationships between the five themes are not clear and there is a lack of an "overarching" theme for them. Now the five themes do not support a complete theme at a higher level. I think this is a key issue in data analysis. The word "the" between "led" "to" in the last sentence is redundant.
3. Weaknesses: I do not see the purposeful sampling as a weakness for a qualitative study. Instead it is appropriate if the authors did it correctly.
4. Introduction: Please provide the context of critical care in Tanzania, with what has known in similar low-income countries, leading to the problem, the rationale of the study and the aim of study. Now the context and background are missing.
5. The study aim should be made clearer with specific objectives: the lessons learned from applying EECC in the country refer to the barriers and facilitators of the complex implementation.
6. Methods: The data analysis was not conducted on transcription, which would be a main limitation to the study quality. It is unclear how themes were developed from codes predetermined in a codebook. If an existing method was used or adapted, reference of the publications is needed.
7. Results-Participants: the characteristics of participants were insufficient with no ages, education levels, years of working experience, etc.
8. Results-Themes: The third theme has numbering subthemes. The last theme is much smaller than others. Each theme has a short paragraph describing or defining the brief content, and others are the quotes. I think all themes are not sufficiently powerful. Another key issue is that the five theme have no logical links between each other to bond them into a whole. I think mapping the codes into an implementation theory might be useful during analysis, such as NPT. In addition, [Participant number 5] ending each quote does not make sense to the readers. I would use something like this: [Policy maker, Female, 36] (profession, gender, age)
9. Discussion: this is brief and only three themes have been discussed? The discussion didn't suggest that it is a complex process of implementing EECC in the country.
10. Limitations: Again, purposively selecting participants and the "subjectivity" are not limitations of a qualitative study.
11. Conclusion: The findings of the study cannot be generalized to the worldwide (globally used by the authors). The implications of a qualitative study are to transfer the themes into similar context

to the setting of the study, Tanzania.

VERSION 1 - AUTHOR RESPONSE

Reviewer 1

Comment 1: Dear authors, thank you very much for the opportunity to review the submitted manuscript. The manuscript includes a qualitative study evaluating an improvement project in Tanzania. I am appreciating this well-written work and have got some minor concerns.

Thank you for your positive and thoughtful feedback.

Comment 2: Page 5 (Abstract), Line 24: since this is a qualitative research, please use the term "Findings" instead of "Results"; and in Conclusions "the findings from this study
…"; and in main manuscript.

Thank you, we have replaced the term "Results" with "Findings" as advised.

Comment 3: Page 7, Data analysis: please add a reference and method to this paragraph.

Thank you, we have added this.

Comment 4: P12: in the first paragraph in section Discussion, the design, method, and main findings are summarized. The discussion incl references etc. starts in the second paragraph.

Thanks for this comment. The discussion has been re-arranged, and the first paragraph now contains more discussion and references.

Reviewer 2

Comment 1 - Title: I think it is too long, with two colons. Remove "Improving the care of critically ill patients:"

Response: Thank you for this suggestion. We have revised the title as suggested.

Comment 2 - Abstract: I cannot agree with the authors' Conclusions on the Results that it is a "holistic, comprehensive approach" because the relationships between the five themes are not clear and there is a lack of an "overarching" theme for them.

Now the five themes do not support a complete theme at a higher level. I think this is a key issue in data analysis. The word "the" between "led" "to" in the last sentence is redundant.

Response: We appreciate this feedback. We have made changes to the methods, results and abstract to address this point, including a mapping to the NPT framework (see other responses). We have also corrected the sentence in the abstract by removing the redundant word "the."

Comment 3 - Weaknesses: I do not see the purposeful sampling as a weakness for a qualitative study. Instead it is appropriate if the authors did it correctly.

Response: Thank you for this observation. We have removed purposeful sampling from the weaknesses.

Comment 4 - Introduction: Please provide the context of critical care in Tanzania, with what has known in similar low-income countries, leading to the problem, the rationale of the study and the aim of study. Now the context and background are missing.

Response: Thank you for this observation. We have revised the introduction to include more context.

Comment 5 - The study aim should be made clearer with specific objectives: the lessons learned from applying EECC in the country refer to the barriers and facilitators of the complex implementation.

Response: We have revised the aim to be more specific and emphasized that the lessons reflect identified barriers and facilitators to EECC implementation.

Comment 6 - Methods: The data analysis was not conducted on transcription, which would be a main limitation to the study quality. It is unclear how themes were developed from codes predetermined in a codebook. If an existing method was used or adapted, reference of the publications is needed.

Response: Thank you for this comment. We have now acknowledged in the Strengths and Limitations section the absence of transcription and translation as the main limitation of the study. We have also clarified in the Methods – Data Analysis section that the codes were grouped into categories, from which overarching themes were identified.

Comment 7 - Results-Participants: the characteristics of participants were insufficient with no ages, education levels, years of working experience, etc.

Response: Thank you. We have revised the Participants section to include the missing participant characteristics.

Comment 8: Results-Themes: The third theme has numbering subthemes. The last theme is much smaller than others. Each theme has a short paragraph

describing or defining the brief content, and others are the quotes. I think all themes are not sufficiently powerful. Another key issue is that the five themes have no logical links between each other to bond them into a whole. I think mapping the codes into an implementation theory might be useful during analysis, such as NPT. In addition, [Participant number 5] ending each quote does not make sense to the readers. I would use something like this: [Policy maker, Female, 36] (profession, gender, age)

Response: We appreciate this feedback. In response, we have:

- Revised the presentation of Theme 3 to remove numbered subthemes
- Expanded Theme 5 more to align better with the others
- Mapped the five themes to the four domains of Normalization Process Theory (NPT)
- Revised all quote attributions to follow the format recommended

Comment 9: Discussion: this is brief and only three themes have been discussed? The discussion didn't suggest that it is a complex process of implementing EECC in the country.

Response: Thank you for this comment. We have revised the discussion section to include all the five themes and frame the process of implementing EECC as complex.

Comment 10: Limitations: Again, purposively selecting participants and the "subjectivity" are not limitations of a qualitative study.

Response: We have removed these points from the limitations section.

Comment 11: Conclusion: The findings of the study cannot be generalized to the worldwide (globally used by the authors). The implications of a qualitative study are to transfer the themes into similar context to the setting of the study, Tanzania.

Response: Thank you for this observation. We have revised the Conclusion.
